# The cutaneous mirror: leveraging drug-induced skin phenotypes as early visual risk signals for systemic toxicity, a comprehensive review

**DOI:** 10.3389/fdsfr.2026.1825005

**Published:** 2026-06-11

**Authors:** Hind B. Alshalhoob, Lama M. Albelowi, Aseel S. Alotaibi, Shada Khalid Alanazi, Leen E. Alturki, Zahra Saleh Alsindi, Waad Abdulelah Alduraywish, Sarah Anwar Almulla, Ahmed Anwar Almulla, Nouf Abdulaziz Almagushi, Sarah Khalifah Alkhezzi

**Affiliations:** 1 College of Medicine, Majmaah University, Al Majma’ah, Saudi Arabia; 2 Dermatology Department, King Salman Bin Abdulaziz Medical City, Madinah, Saudi Arabia; 3 College of Medicine, King Faisal University, Al Ahsa, Saudi Arabia; 4 Ministry of Health, Riyadh, Saudi Arabia; 5 Dermatology Department, King Fahd Specialist Hospital, Buraydah, Saudi Arabia

**Keywords:** adverse drug reactions, clinical drug safety, drug eruptions, pharmacovigilance, risk stratification, severe cutaneous adverse reactions, systemic toxicity

## Abstract

Drug-induced cutaneous phenotypes can act as early, bedside-visible clinical risk signals of systemic toxicity because the skin externalizes immune dysregulation, epithelial injury, and microvascular disturbance before organ-specific symptoms are obvious. This narrative review synthesizes peer-reviewed human evidence linking drug-related eruptions to systemic harm and actionable clinical decisions. We searched databases through January 2026 with citation chasing and organized findings using a morphology-anchored framework cross mapped to drug classes and mechanisms. High-risk patterns repeatedly signal urgent systemic risk: painful dusky or targetoid lesions with mucosal involvement and blistering/epidermal detachment require immediate culprit withdrawal and admission-level supportive care; widespread eruption with facial edema plus fever, lymphadenopathy, eosinophilia, or atypical lymphocytosis requires drug discontinuation and close monitoring for drug reaction with eosinophilia and systemic symptoms (DRESS). Research priorities include prospective validation of phenotypes as quantitative predictive risk signals, harmonized outcomes, reproducible imaging standards, and bias-aware digital implementation.

## 1 Introduction

The skin is uniquely positioned to act as a sentinel organ for drug toxicity because it is continuously exposed, densely populated with immune sentinels, and designed to translate chemical and inflammatory danger signals into visible morphologic change (Nestle et al., 2009). Skin immunosurveillance relies on coordinated sensing and effector functions across keratinocytes, antigen-presenting cells, resident T cells, and vascular and lymphatic networks, creating an early warning system that can externalize internal immune dysregulation and toxic injury (Kupper and Fuhlbrigge, 2004; Di Meglio et al., 2011). In many systemic disorders, the surface phenotype provides actionable clues that prompt diagnostic reorientation and urgent referral, and the same principle applies to medication harms, where the first clinically detectable sign may be cutaneous rather than visceral (Marzano et al., 2016; Sampaio et al., 2021).

Drug-induced skin phenotypes are common across inpatient and outpatient practice, spanning benign self-limited rashes to severe cutaneous adverse reactions (SCARs) with multiorgan involvement and high mortality risk (Del Pozzo-Magaña and Liy-Wong, 2024; Lee et al., 2010). Importantly, the skin can signal systemic toxicity through recognizable patterns that track with immune activation, epithelial injury, vascular inflammation, or drug accumulation, and these patterns may precede laboratory abnormalities or organ-specific symptoms (Marzano et al., 2016; Zalewska-Janowska et al., 2017). SCARs illustrate this sentinel role most clearly. Drug reaction with eosinophilia and systemic symptoms (DRESS) is defined by a heterogeneous eruption coupled to internal organ involvement, often hepatic, renal, pulmonary, or cardiac, and diagnostic frameworks such as RegiSCAR were developed because clinical recognition is time sensitive and complex (Sasidharanpillai et al., 2022; Calle et al., 2023). Stevens–Johnson syndrome and toxic epidermal necrolysis (SJS–TEN) represent mucocutaneous epidermal necrosis with systemic illness and substantial mortality, where early recognition and supportive management are central (Shah H. et al., 2024). Acute generalized exanthematous pustulosis (AGEP) is classically pustular and febrile and can include systemic involvement in a clinically meaningful subset, reinforcing that a surface eruption can be a systemic event (Szatkowski and Schwartz, 2015).

Visual risk signals matter because they can accelerate triage, shorten time to culprit drug withdrawal, and prevent progression to severe outcomes. In epidermal necrolysis, time to stopping the offending medication is linked to survival, making the skin finding not merely descriptive but operational for risk reduction (Miliszewski et al., 2016). Beyond classic hypersensitivity syndromes, oncology has made the clinical value of visible toxicity especially clear. Cutaneous reactions during epidermal growth factor receptor inhibition, such as acneiform eruptions, can reflect on-target pathway inhibition and have been repeatedly associated with treatment activity, making the skin phenotype a real-time pharmacodynamic readout that can guide supportive care and dosing decisions (Urban and Anadkat, 2013). Likewise, immune checkpoint inhibitors frequently cause early cutaneous immune-related adverse events, and these visible toxicities can serve as the first clinical signal of systemic immune activation, warranting closer monitoring for extracutaneous immune toxicities (Wang et al., 2021; Apalla et al., 2021; Vaez-Gharamaleki et al., 2025).

This review focuses on drug-induced skin phenotypes as early visual risk signals that can signal systemic toxicity, with priority given to patterns that predict organ involvement, clinical deterioration, or the need for urgent drug cessation and escalation of care. While individual phenotype–drug associations have been described in prior reviews, this work adds value by integrating morphology-anchored pattern recognition with an explicit risk-stratification framework that separates high-risk, time-critical phenotypes from lower-risk or self-limited reactions, providing a clinically actionable triage pathway supported by a minimum laboratory set and clear escalation criteria. In this review, a skin phenotype refers to a reproducible, clinician-observable pattern of cutaneous change defined by morphology, distribution, timing relative to drug exposure, and accompanying mucosal, appendageal, or vascular features (Del Pozzo-Magaña and Liy-Wong, 2024). Systemic toxicity refers to drug-related injury or dysfunction affecting internal organs or whole-body physiology, including immune dysregulation, epithelial injury, and organ inflammation, whether clinically apparent or detectable by laboratory or imaging abnormalities (Marzano et al., 2016). An early warning sign refers to a cutaneous phenotype that appears early enough in the toxicity trajectory to permit timely intervention that changes risk, most often by stopping the offending agent, modifying exposure, or initiating targeted treatment (Lee et al., 2010). On-target toxicity refers to adverse effects arising from exaggerated pharmacologic action at the intended target in tissues that share that target biology, while off-target toxicity refers to adverse effects mediated through unintended targets or non-specific chemical injury pathways, concepts used broadly in toxicology and translational safety science (Rudmann, 2013; Garon et al., 2017). Finally, immune-mediated toxicity refers to reactions driven by adaptive or innate immune mechanisms, such as drug-specific T-cell responses and related cytokine programs, whereas direct toxicity refers to nonimmune injury, including cumulative toxicity, photosensitivity, and other nonimmunologic mechanisms that still produce distinctive skin phenotypes (Lee, 2024).

## 2 Methods

This narrative review was developed using a structured search combined with iterative citation chasing, aligning with published recommendations for improving transparency and reproducibility in narrative reviews and with key quality domains captured by the Scale for the Assessment of Narrative Review Articles (SANRA) (Baethge et al., 2019). We searched PubMed, Embase, Web of Science, Scopus, and the Cochrane Central Register of Controlled Trials from database inception through January 2026. Search terms were combined across three concept families: drug exposure, cutaneous phenotype, and systemic toxicity signal, using keywords such as cutaneous adverse drug reaction, drug eruption, drug hypersensitivity, skin toxicity, severe cutaneous adverse reaction, SJS–TEN, DRESS, AGEP, vasculitis, and biomarker or signal. Eligible records were peer-reviewed human studies that described a temporally linked, drug-associated skin phenotype with documented systemic toxicity, organ involvement, clinically significant physiologic derangement, hospitalization, or a clear need for urgent drug withdrawal or escalation of care. We excluded purely local irritant reactions without systemic relevance, non-drug etiologies, and articles without sufficient clinical detail to assign a phenotype or link it to systemic outcomes.

Phenotypes were organized primarily using a morphology-anchored framework because bedside recognition typically begins with pattern identification, distribution, timing, and mucosal involvement, rather than with prior certainty about drug class (Brockow et al., 2019). This morphology-centered approach was cross mapped to drug classes and mechanistic groupings to support clinical triage and capture recurring syndrome-level entities, particularly severe reactions where early recognition and prompt withdrawal of the causative drug are central to the outcome. Classification decisions were informed by contemporary summaries of cutaneous adverse drug reaction patterns and by consensus frameworks for drug hypersensitivity and SCARs (Del Pozzo-Magaña and Liy-Wong, 2024). When a phenotype could be reasonably attributed to more than one category, for example, overlap between exanthematous eruptions and early severe cutaneous adverse reactions, we privileged the higher risk categorization when systemic toxicity features were present or when the literature described a progression risk (Pinto Gouveia et al., 2016).

Evidence was appraised using a light-touch hierarchy appropriate for harms, recognizing that drug safety signals often originate outside randomized trials and that different evidence streams contribute distinct strengths in pharmacovigilance. Randomized trials and comparative observational studies were used to inform incidence, risk modifiers, and treatment associations when available, while acknowledging broader debates about hierarchies of evidence across designs. Pharmacovigilance sources, including spontaneous report analyses and disproportionality methods, were used to identify signal patterns, detect rare events, and generate drug phenotype associations that may not be visible in trials. Case reports and case series were included to capture rare, high-acuity phenotypes and early clinical warning patterns, and they were interpreted with attention to reporting completeness using established case reporting guidance. Throughout the review, qualifying language is used to indicate the type and strength of supporting evidence. Recommendations supported primarily by case series and pharmacovigilance data are distinguished from those informed by comparative studies or consensus guidelines, and expert opinion is identified where evidence is limited or absent.

## 3 The skin as a window to systemic harm

The skin can reveal systemic harm early because it sits at the interface of chemical exposure and host defense, and because its resident cells translate danger signals into visible changes. Keratinocytes are not passive barrier bricks (Nguyen and Soulika, 2019). They sense injury and microbial patterns, activate innate programs, and coordinate cytokine and chemokine signaling that recruit and shape immune responses, converting molecular toxicity into a readable eruption (Pavlos et al., 2015). The skin also contains a dense network of resident T cells and antigen-presenting cells that can mount delayed drug hypersensitivity responses with systemic consequences, making early surface findings clinically meaningful rather than cosmetic (Pavlos et al., 2015; Thomson et al., 2022).

Microvascular biology provides a second early detection channel. The cutaneous circulation is a high-signal tissue in which disturbances in vasomotor tone, endothelial activation, permeability, and occlusion can rapidly produce patterned discoloration, purpura, retiform changes, or necrosis (Munoz et al., 2020; Sajjan et al., 2015). This matters for drug toxicity because many severe adverse reactions involve endothelial injury and coagulation imbalance, and the skin is one of the first organs where small vessel dysfunction becomes visible (Thornsberry et al., 2013). Epidermal renewal also contributes to sensitivity. Keratinocytes continuously balance self-renewal and differentiation, with rapid turnover that makes the epidermis vulnerable to cytotoxic injury, metabolic disruption, and inflammatory derailment (Finnegan et al., 2019).

Across drug classes, several shared pathways link a phenotype to systemic risk. Delayed drug hypersensitivity is commonly driven by T-cell mechanisms and can progress from a morbilliform eruption to severe syndromes with hepatic, renal, pulmonary, hematologic, or cardiac involvement. The skin may be the first visible proxy for a whole-body immune crisis (Pichler, 2025; Shah P. N. et al., 2024). Vasculitis provides another bridge. Drug-induced small-vessel vasculitis often presents with palpable purpura and can remain skin limited or progress to systemic disease with kidney, gastrointestinal, joint, or constitutional involvement. The rash could serve as an entry point for organ risk stratification (Castañeda et al., 2024; Kelly et al., 2022). Thrombotic and vasculopathic pathways can also be externally legible. Reticular or racemose livedo patterns and retiform purpura reflect impaired cutaneous blood flow and are strongly linked to systemic hypercoagulable and thromboembolic states, making the skin a practical screening surface for potentially life-threatening vascular risk (Kontic et al., 2018; Lamadrid-Zertuche et al., 2018). Mitochondrial toxicity and direct cytotoxicity illustrate nonimmune routes. Mitochondrial dysfunction is a recognized mechanism in drug-induced organ injury. It can also intersect with cutaneous and adnexal biology, while cytotoxic agents can injure rapidly renewing epithelia and eccrine-rich sites, producing phenotypes such as hand-foot syndrome that often track cumulative exposure and may force treatment modification (Kwakman et al., 2020; Natarelli et al., 2024; Begriche et al., 2011).

**FIGURE 1:**
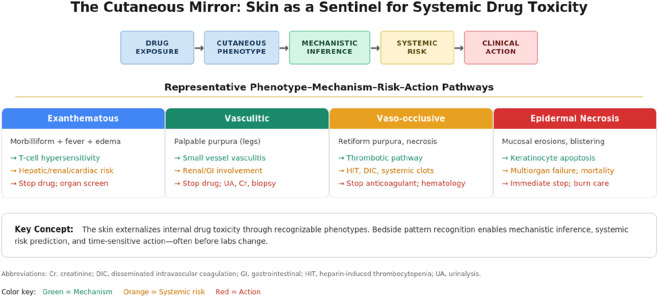
Cutaneous mirror model linking drug exposure to systemic toxicity.

A practical conceptual model for this review is phenotype → mechanism → systemic risk → action. A patterned eruption becomes clinically useful when it reliably maps to a small set of mechanisms, and those mechanisms predict a bounded set of systemic hazards that require specific steps. Reviews that connect cutaneous adverse drug reactions to organ damage support this mechanistic mapping approach, particularly when time to drug withdrawal and targeted evaluation changes outcomes (Marzano et al., 2016; Del Pozzo-Magaña and Liy-Wong, 2024). In this framework, an early exanthem accompanied by systemic symptoms signals immune-mediated hypersensitivity and triggers prompt cessation of the causative drug and screening for organ involvement (Stirton et al., 2022; Broyles et al., 2020). Palpable purpura signals small vessel inflammation and directs evaluation for systemic vasculitis features, including renal and gastrointestinal involvement, while reticular livedo patterns signal flow disturbance and prompt assessment for thrombotic drivers and urgent prevention of progression to necrosis or systemic thrombosis (Sajjan et al., 2015; Fraticelli et al., 2021). Hand-foot syndrome and other cytotoxic injury phenotypes signal dose-limiting epithelial damage and support early supportive care and treatment adjustment to prevent escalation and preserve systemic therapy delivery (Shar et al., 2013; Vargas-Hernández and Vargas Aguilar, 2024). The conceptual framework linking cutaneous phenotypes to mechanistic inference, systemic risk, and clinical action is summarized in Figure 1.

## 4 Morphology-based taxonomy of drug-induced skin phenotypes

Each phenotype syndrome is described systematically using a structured format, including visual appearance, associated symptoms or their absence, and typical latency relative to drug exposure.

### 4.1 Exanthematous and urticarial patterns

#### 4.1.1 Morbilliform eruption

Visual appearance consists of diffuse pink to red macules and papules that coalesce into patches, often starting on the trunk and spreading symmetrically, typically sparing mucosa in uncomplicated cases (Yan et al., 2025; Ernst and Giubellino, 2022). Common causative drugs include beta-lactam antibiotics, sulfonamides, antiepileptics, allopurinol, and many other agents used in the inpatient setting, with morphology alone insufficient to assign causality without timing and medication history (Noe et al., 2018). Systemic toxicities flagged are usually limited for uncomplicated exanthems, but this pattern can be an early hypersensitivity signal when paired with fever, facial edema, lymphadenopathy, eosinophilia, atypical lymphocytosis, or evolving organ injury, particularly early DRESS (Choudhary et al., 2013; Kardaun et al., 2013). Timing is classically several days to a few weeks after starting the culprit, with longer latency raising suspicion for DRESS when systemic features appear. Red flags include mucosal erosions, skin pain, dusky targets, blistering, facial edema, hypotension, rapidly progressive rash, laboratory signals of hepatitis, nephritis, cytopenias, or myocarditis, and evolution or overlap with a higher risk phenotype such as epidermal necrolysis, DRESS, or vasculitic or necrotic patterns (Ramirez et al., 2023; Dagnon da Silva et al., 2023). Workup should prioritize basic severity screening rather than exhaustive testing in every patient, focusing on complete blood count with differential, liver enzymes, creatinine, and urinalysis when systemic symptoms or high-risk drugs are present, and dermatology consultation plus biopsy when features suggest a SCAR or vasculitis (Yan et al., 2025). Management is to stop the most likely culprit if red flags are present, avoid rechallenge, provide symptomatic care for low-risk cases, and escalate to urgent SCAR evaluation when mucosa, skin pain, blistering, or systemic instability appear (Yan et al., 2025; Chang et al., 2022).

#### 4.1.2 Urticaria and angioedema

The visual appearance consists of transient wheals with pruritus and blanching, sometimes with deeper swelling of lips, eyelids, tongue, or airway (Kanani et al., 2018). Common culprits include antibiotics, nonsteroidal anti-inflammatory drugs, radiocontrast, and many biologics, with angiotensin-converting enzyme inhibitors a key trigger for bradykinin-mediated angioedema that may lack hives (Pier and Bingemann, 2020; Puxeddu et al., 2016). Systemic toxicities flagged range from none to immediate anaphylaxis risk, so the phenotype becomes a systemic warning sign when accompanied by dyspnea, stridor, hypotension, syncope, or gastrointestinal symptoms suggestive of systemic mediator release (Hanschmann et al., 2023). Timing is often minutes to hours after exposure for immediate reactions, although delayed urticaria can occur and should be interpreted alongside exposure chronology (Kanani et al., 2018). Red flags include airway symptoms, rapidly progressive tongue or laryngeal swelling, hypotension, and refractory symptoms despite first-line therapy (Bizjak et al., 2025). Workup is mostly clinical for acute events, with targeted evaluation for bradykinin-mediated angioedema when hives are absent and for suspected drug allergy documentation to prevent future high-risk re-exposure (Nettis et al., 2020). Management is to treat suspected anaphylaxis immediately with intramuscular epinephrine and supportive care, stop the suspected trigger, avoid angiotensin-converting enzyme inhibitors permanently in bradykinin angioedema, and arrange allergy evaluation when drug allergy labeling will affect future care (Miller et al., 2019; Macy, 2021).

#### 4.1.3 Serum sickness-like patterns

Visual appearance combines urticarial or morbilliform eruptions with fever and prominent arthralgia or arthritis, sometimes with lymphadenopathy, and can be confused with viral illness or an evolving SCAR (Chatzigrigoriadis et al., 2025; Khalaf et al., 2025). Common causative drugs include beta-lactam antibiotics such as cefaclor and amoxicillin, along with other drugs and occasional vaccine exposures (Steering Committee Authors et al., 2025). Systemic toxicities flagged are usually inflammatory and self-limited but can drive significant functional impairment, prolonged symptoms, and diagnostic error that delays stopping the trigger (Rixe and Tavarez, 2025). Timing is typically days to weeks after exposure, with recurrence after re-exposure supporting the diagnosis (Khalaf et al., 2025). Red flags include mucosal erosions, skin pain, blistering, hypotension, or laboratory evidence of organ injury that would redirect concern toward a SCAR (Cho and Chu, 2017). Workup is guided by severity and differential diagnosis, commonly including complete blood count, inflammatory markers, and renal and hepatic function when systemic symptoms are significant (Chatzigrigoriadis et al., 2025). Management is to stop the culprit drug, provide symptomatic therapy for pain and pruritus, and document the reaction to prevent re-exposure, with specialist input when the clinical picture overlaps with SCARs (Jeimy et al., 2025).

### 4.2 Purpura, vasculitic, and livedoid patterns

#### 4.2.1 Palpable purpura and cutaneous small vessel vasculitis

Visual appearance consists of non-blanching purple papules and plaques, often on dependent areas such as the legs, sometimes with burning or pain (Alpsoy, 2022). Common culprit drugs include beta-lactam antibiotics and nonsteroidal anti-inflammatory drugs, among many others, and the skin-limited form can still signal a broader systemic vasculitis in a subset (Al-Nesf et al., 2013). Systemic toxicities flagged include renal involvement, gastrointestinal involvement, arthralgias, and constitutional symptoms, with drug-induced disease capable of being either skin limited or systemic. Timing often occurs days to weeks after exposure, and recurrence after re-exposure strengthens causality (Russell and Gibson, 2006). Red flags include hematuria, proteinuria, abdominal pain, melena, neurologic symptoms, rapidly progressive purpura, or necrosis (Alpsoy, 2022). Workup generally includes skin biopsy with direct immunofluorescence when diagnosis is uncertain or severe, plus urinalysis and renal function assessment to detect systemic involvement (Alpsoy, 2022; Chango Azanza et al., 2020). Management is to stop the suspected drug when temporally plausible, assess for systemic involvement, and coordinate rheumatology or nephrology input when renal or gastrointestinal features appear (Russell and Gibson, 2006).

#### 4.2.2 Retiform purpura, livedo racemosa, and cutaneous necrosis

Visual appearance is angulated or netlike purpura that may progress to necrosis, reflecting vessel occlusion or severe vascular injury rather than simple capillary leakage (Georgesen et al., 2020; Timoney et al., 2019). Common culprits and contexts include anticoagulant-related necrosis syndromes, heparin-induced thrombocytopenia with skin necrosis, vasopressor-related ischemia, and drug-triggered coagulopathies, with pattern recognition guiding urgent systemic evaluation (Dissemond et al., 2024). Systemic toxicities flagged include thrombotic syndromes, antiphospholipid spectrum disease, disseminated intravascular coagulation in sepsis, and catastrophic vascular occlusion, all of which carry immediate morbidity and mortality risk (Wysong and Venkatesan, 2011; Abdul Gafoor et al., 2026; Patriarcheas et al., 2025). Timing is often tightly linked to the inciting exposure, such as early days after starting warfarin for warfarin-induced skin necrosis, or several days after heparin exposure for heparin-induced thrombocytopenia-associated necrosis, although distant site necrosis can occur (Kakagia et al., 2014; Quiles-Recuenco et al., 2023). Red flags include severe pain, rapid progression, hemorrhagic bullae, expanding necrosis, fever or shock, and laboratory signs of thrombocytopenia or coagulopathy (Mohamed Osman et al., 2025; David et al., 2024). Workup requires immediate coagulation and platelet assessment, evaluation for heparin-induced thrombocytopenia when appropriate, and broader evaluation for sepsis and antiphospholipid syndrome when the livedoid pattern is widespread or accompanied by thrombosis (Wysong and Venkatesan, 2011; Abdul Gafoor et al., 2026; Tassava and Warkentin, 2015). Management is a stop rule for the offending anticoagulant when suspected, urgent hematology input, initiation of alternative anticoagulation when indicated for heparin-induced thrombocytopenia, reversal and management protocols for warfarin-induced necrosis, and sepsis-directed resuscitation when purpura fulminans is suspected (Abdul Gafoor et al., 2026; Mohamed Osman et al., 2025; David et al., 2024).

### 4.3 Severe cutaneous adverse reactions

#### 4.3.1 Stevens-Johnson syndrome and toxic epidermal necrolysis

Visual appearance is painful erythematous or dusky lesions that progress to blistering and epidermal detachment with prominent mucosal erosions, often accompanied by fever and systemic illness (Miliszewski et al., 2016). Common culprits include high-risk antibiotics, antiepileptics, allopurinol, and other agents known to trigger epidermal necrolysis, with recent drug exposure history central to triage (Shah H. et al., 2024). Systemic toxicities flagged include fluid loss, electrolyte disturbance, infection risk, respiratory compromise, and multiorgan failure, with high mortality in toxic epidermal necrolysis (TEN) (Canhão et al., 2022). Timing is commonly within the first weeks after starting a new medication, and the earliest actionable signal may be a prodrome with mucosal pain and skin tenderness before full detachment (Harris et al., 2016). Red flags include skin pain out of proportion to rash, any mucosal erosions, blistering, targetoid dusky lesions, and rapid progression. Workup includes immediate severity assessment using validated mortality tools such as SCORTEN, baseline labs to guide intensive supportive care, infection surveillance, and early specialty involvement (Charlton et al., 2020). Management is an immediate stop rule for all potential culprit drugs, urgent admission, burn-unit-style supportive care, and multidisciplinary management, because prompt withdrawal of the causative drug is linked to improved survival (Al-Benna, 2021).

#### 4.3.2 Drug reaction with eosinophilia and systemic symptoms

Visual appearance is a widespread eruption that may be morbilliform, edematous, or polymorphic, frequently accompanied by facial edema and systemic symptoms such as fever and malaise (Stirton et al., 2022). Common culprits include anticonvulsants, allopurinol, sulfonamides, and other agents, with a characteristic longer latency that helps distinguish it from immediate allergy (Soria et al., 2021; Woodruff and Botto, 2022). Systemic toxicities flagged include hepatitis, nephritis, pneumonitis, cytopenias, and, less commonly, myocarditis that can be fatal (Watanabe et al., 2024; Oliveira et al., 2016). Timing often occurs 2 weeks to 8 weeks after drug initiation, which is a key bedside clue when a patient deteriorates weeks into therapy (Calle et al., 2023). Red flags include jaundice, rising transaminases, creatinine increase, dyspnea, chest pain, tachycardia, syncope, and rapidly progressive systemic symptoms (Castellazzi et al., 2018). Workup should be systematic rather than symptom driven because organ involvement may be clinically silent early, typically including complete blood count with differential, liver enzymes, bilirubin, creatinine, urinalysis, and targeted cardiac evaluation when symptoms or biomarker abnormalities suggest involvement (Cho et al., 2017). Management is to stop the culprit drug immediately, document avoidance, initiate close monitoring for organ involvement over weeks, and escalate immunosuppression decisions with specialist input when internal organ injury progresses (Wang et al., 2024).

#### 4.3.3 Acute generalized exanthematous pustulosis

Visual appearance consists of a sudden eruption of many small non-follicular pustules on an erythematous base, often with fever and leukocytosis, followed by desquamation (Moore et al., 2025). Common culprits include antibiotics and other acute exposures, with a rapid onset after drug exposure often helping to narrow the culprit list (Que et al., 2025). Systemic toxicities flagged are usually limited, but systemic involvement can occur and requires recognition when fever, organ dysfunction, or hemodynamic instability appear (Feldmeyer et al., 2016). Timing is often rapid, frequently within days of exposure, and resolution is expected within about 2 weeks after withdrawal in typical cases (Que et al., 2025). Red flags include hypotension, extensive skin involvement with pain, mucosal erosions that suggest alternative diagnoses, and laboratory evidence of organ injury. Workup includes confirmation support with histopathology when uncertain and laboratory screening for systemic involvement when clinically indicated (Tetart et al., 2024). Management is to stop the suspected trigger, provide supportive care, and escalate care when systemic involvement is present, or diagnosis overlaps with SJS and TEN (Tetart et al., 2024).

### 4.4 Bullous and blistering phenotypes beyond SJS and TEN

#### 4.4.1 Drug-induced bullous pemphigoid and pemphigus spectrum

Visual appearance is tense pruritic bullae on erythematous or urticarial bases in pemphigoid, and flaccid bullae and erosions in pemphigus, with variable mucosal involvement depending on phenotype (Verheyden et al., 2020). Common culprit drugs include dipeptidyl peptidase 4 inhibitors, immune checkpoint inhibitors, and other medications reported across autoimmune blistering disease literature, with drug-induced forms sometimes improving after withdrawal (Kardaun et al., 2022). Systemic toxicities flagged are less about direct multiorgan failure and more about infection risk, fluid and protein loss, and adverse effects from systemic corticosteroids or steroid-sparing immunosuppression used for control (Didona et al., 2023). Timing can vary widely, from weeks to months after starting the culprit, making medication reconciliation over longer windows important (Verheyden et al., 2020). Red flags include extensive blistering with systemic symptoms, ocular involvement suggesting mucous membrane pemphigoid, and secondary infection signs (Daniel and Murrell, 2019). The workup includes lesional and perilesional biopsies with direct immunofluorescence to confirm autoimmune blistering disease and guide immunosuppressive strategy (Didona et al., 2023). Management is to stop the suspected drug when feasible, start appropriate wound care and anti-inflammatory therapy tailored to severity, and coordinate monitoring for complications of immunosuppression (Myers and Culton, 2025).

#### 4.4.2 Drug-induced linear IgA bullous dermatosis

The visual appearance consists of tense vesicles and bullae that can form annular or clustered patterns and may involve mucosa (Garel et al., 2019). The common culprit drug is vancomycin, with reported onset ranging from about a day to 2 weeks after starting therapy in many cases (Peake and Chow, 2025). Systemic toxicities flagged depend on the clinical context, such as a serious infection requiring vancomycin, so the skin finding becomes a safety signal that forces antimicrobial reassessment and balancing of infection control against immune blistering risk (Yang et al., 2025). Timing is often close to exposure and may persist after discontinuation in patients with renal impairment, supporting the need for careful medication review and renal context (Didona et al., 2023). Red flags include mucosal involvement, extensive blistering, and secondary infection (Starzyk et al., 2024). The workup mirrors other autoimmune blistering diseases, including biopsy and direct immunofluorescence to confirm linear IgA deposition (Lammer et al., 2019). Management is to stop vancomycin when possible, switch to alternative antimicrobials guided by infection needs, and treat the dermatosis severity with dermatology support (Khan et al., 2023).

### 4.5 Pigmentary, nail, and hair changes as toxicity signals

#### 4.5.1 Hyperpigmentation and dyschromia

Visual appearance ranges from blue-gray pigmentation in photo-distributed areas to diffuse or localized hyperpigmentation involving skin, mucosa, and sclera, depending on the drug and pigment deposition pattern (Giménez García and Carrasco Molina, 2019). Common culprits include minocycline, amiodarone, antimalarials, and several chemotherapies, with some patterns linked to cumulative dose and prolonged exposure (Fiscus et al., 2014; Sethumadhavan et al., 2024; Konda et al., 2025). Systemic toxicities flagged are context dependent, sometimes representing drug accumulation that can also involve deeper tissues, sometimes indicating photosensitization behavior that increases chronic photodamage risk, and sometimes serving mainly as a marker of chronic exposure that prompts medication reassessment (Camayo et al., 2025). Timing is typically subacute to chronic over months to years for many pigmentary toxicities, although earlier onset can occur with certain agents (Tang et al., 2025). Red flags include rapid onset with systemic symptoms that would suggest vasculitis or necrosis rather than deposition, and mucosal ulceration, which redirects toward a SCAR or an infection (Shah H. et al., 2024). Workup is often clinical, with biopsy reserved for diagnostic uncertainty, and medication timeline review is the main diagnostic tool (Giménez García and Carrasco Molina, 2019). Management is to confirm the culprit exposure, counsel on sun protection when photosensitization is contributing, and discontinue or substitute the drug when the risk–benefit evaluation is unfavorable, recognizing that reversal may be slow or incomplete. New laser therapy options are emerging (Zhao et al., 2023).

#### 4.5.2 Nail changes and alopecia

Visual appearance includes melanonychia, leukonychia, Beau lines, onycholysis, and nail plate fragility, while hair toxicity can present as diffuse shedding or patterned alopecia depending on the mechanism and drug class (Piraccini and Alessandrini, 2013). Common culprits include cytotoxic chemotherapy and targeted therapies, with nail matrix injury producing characteristic transverse or longitudinal changes that can track dose cycles (Samal et al., 2021). Systemic toxicities flagged vary, sometimes reflecting marrow toxicity context in oncology care, sometimes reflecting nutritional compromise from systemic illness, and often functioning as an adherence-limiting toxicity signal that can precede dose reductions or interruptions (Trivedi et al., 2024; Gilbar et al., 2009). Timing aligns with growth kinetics, so nails often show changes weeks after exposure, while chemotherapy-related alopecia typically starts within weeks of treatment initiation (Valeyrie-Allanore et al., 2007). Red flags include painful paronychia with systemic signs of infection in immunosuppressed patients and nail bed hemorrhage patterns suggesting thrombocytopenia or coagulopathy in the right clinical context (Piraccini and Alessandrini, 2013). Workup is usually clinical, with blood counts and infection evaluation driven by oncology status and symptoms rather than the nail finding alone (Wollina and Abdel-Naser, 2020). Management centers on supportive care to prevent secondary infection and preserve therapy delivery, plus targeted evaluation when nail findings coincide with systemic cytopenia risk (Patel and Tosti, 2014).

### 4.6 Photosensitivity and phototoxic or photoallergic reactions

Visual appearance ranges from exaggerated sunburn-like erythema, edema, and blistering in phototoxicity to eczematous pruritic dermatitis in photoallergy, usually in photo-exposed distribution (Di Bartolomeo et al., 2022; Rok et al., 2024). Common culprits include tetracyclines, thiazide diuretics, amiodarone, voriconazole, and several oncology drugs, with systematic reviews cataloging frequent offenders in clinical reports (Hofmann and Weber, 2021). Systemic toxicities flagged are most often chronic photodamage risk and, for selected agents such as voriconazole in transplant recipients, elevated cutaneous squamous cell carcinoma risk that makes photosensitivity a long-term safety signal rather than a transient nuisance (Di Bartolomeo et al., 2022). Timing can be immediate with sun exposure after starting a photosensitizer or cumulative over weeks, and recurrence with re-exposure is common (Alrashidi et al., 2020). Red flags include blistering with systemic symptoms suggesting broader drug reaction, extensive involvement with dehydration risk, and persistent phototoxicity in immunosuppressed patients where cancer risk is elevated (Davis et al., 2024). Workup is usually clinical, with phototesting or biopsy reserved for uncertainty, and careful medication and sun-exposure history being the key diagnostic step (Hofmann and Weber, 2021). Management is strict photoprotection and medication reassessment, with stronger urgency for voriconazole-exposed transplant populations where cohort data link exposure to increased keratinocyte carcinoma risk (Kim et al., 2018).

### 4.7 Mucocutaneous patterns involving the mouth, eyes, and genitals

Visual appearance includes stomatitis or mucositis with erosions and pain, conjunctivitis or ocular surface inflammation, and genital ulcers or aphthous-like lesions, sometimes as part of broader mucocutaneous syndromes (Villa and Kuten-Shorrer, 2023). Common culprits include cytotoxic chemotherapy, targeted therapies including EGFR inhibitors, and immune checkpoint inhibitors, each with distinct lesion patterns and timelines (Deutsch et al., 2020; Srivastava et al., 2024). Systemic toxicities flagged include neutropenia-associated infection risk in chemotherapy-related mucositis, dehydration and nutritional compromise, and SJS and TEN spectra when mucosal disease accompanies painful skin lesions and epidermal detachment (Naidu et al., 2004). Timing is often within treatment cycles for chemotherapy, variable for targeted therapy, and can occur early in immune checkpoint inhibitor courses, so the timing relative to regimen changes helps interpret risk (Tao et al., 2025). Red flags include the inability to swallow, fever in a neutropenic patient, ocular pain or vision change, extensive genital erosions, and mucosal involvement paired with skin pain or blistering (Elad et al., 2022). Workup depends on context, with immediate blood count assessment and infection evaluation when febrile or immunosuppressed, and ophthalmology evaluation when ocular involvement occurs (Deutsch et al., 2020). Management is supportive care with pain control and oral hygiene measures, rapid neutropenia and infection triage when indicated, and immediate SCAR stop rules when mucosal disease sits within an epidermal necrolysis picture (Brown and Gupta, 2020).

### 4.8 Acneiform eruptions and follicular disorders

Visual appearance consists of papulopustular follicular eruption on the face, scalp, and upper trunk without comedones, often accompanied by xerosis, fissuring, and paronychia in targeted therapy settings (Nair et al., 2025). Common culprits include EGFR inhibitors, systemic corticosteroids, lithium, and halogenated compounds such as iodides, with EGFR inhibitors producing a well-described, on-target cutaneous toxicity (Fabbrocini et al., 2015). Systemic toxicities flagged differ by drug class, with EGFR inhibitor rash serving as a pharmacodynamic exposure marker in many oncology contexts and severe skin toxicity driving dose modifications that can affect cancer therapy delivery (Abdullah et al., 2012). Timing for EGFR inhibitors is often within the first weeks of therapy, which makes early prophylaxis and treatment strategies clinically useful (Abdullah et al., 2012). Red flags include extensive skin breakdown, secondary infection, fever, or inability to maintain therapy due to symptom burden (Lacouture et al., 2011). Workup is usually clinical, with bacterial culture or evaluation for superinfection when pustules crust, pain increases, or systemic symptoms appear (Fabbrocini et al., 2015). Management is to start guideline-informed supportive care early, often with topical anti-inflammatory and antimicrobial strategies and, when indicated, systemic tetracyclines, while coordinating with oncology to preserve effective dosing when feasible (Baas et al., 2012; Gorji et al., 2022).

### 4.9 Injection site and local reactions with systemic implications

**FIGURE 2:**
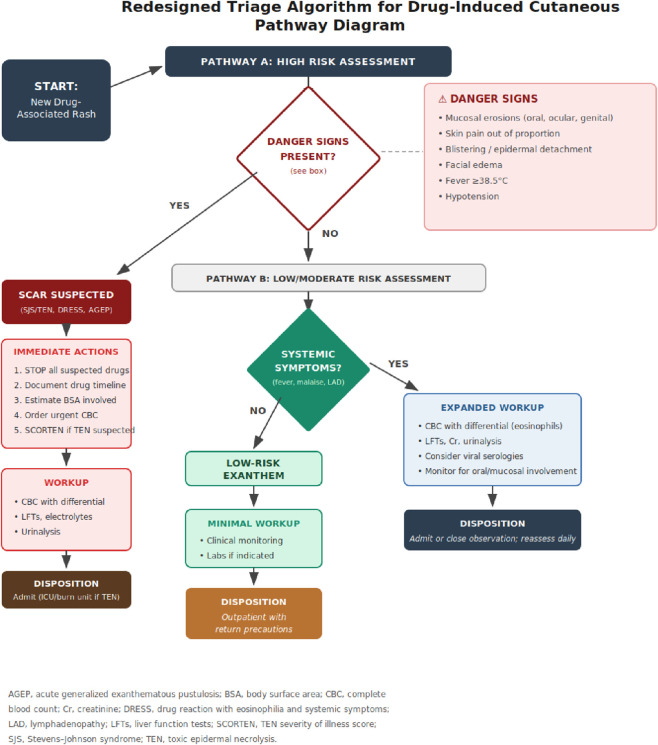
Redesigned triage algorithm for drug-induced cutaneous reactions.

Visual appearance includes immediate erythema, pruritus, and swelling minutes to hours after injection, delayed indurated plaques or eczematous reactions days later, and more concerning patterns such as hemorrhagic purpura or necrosis at or distant from injection sites (Kim et al., 2023). Common culprits include subcutaneously administered biologic therapies, heparins, vaccines, and other injectable agents, with delayed reactions sometimes reflecting T cell-mediated hypersensitivity rather than infection (Zeltser et al., 2001; Patel and Khan, 2017). Systemic toxicities flagged include IgE-mediated immediate allergy with anaphylaxis risk, immune complex vasculitis in selected settings, and heparin-induced thrombocytopenia characterized by painful purpura progressing to necrosis, thrombocytopenia, and thrombosis risk (Fraticelli et al., 2021; Hanschmann et al., 2023). Timing separates phenotypes, with immediate reactions suggesting acute allergy, delayed reactions appearing over one to several days, and heparin-induced necrosis often developing after days of exposure or even at distant sites in heparin-induced thrombocytopenia (Broyles et al., 2020; David et al., 2024). Red flags include systemic symptoms after injection, hypotension or respiratory symptoms, rapidly progressive pain and necrosis, and unexplained thrombocytopenia (Zeltser et al., 2001; Singla et al., 2013). Workup focuses on ruling out anaphylaxis in acute presentations, infection when warmth and tenderness predominate, and urgent platelet and coagulation evaluation when necrosis or retiform purpura appears, with heparin-induced thrombocytopenia testing when clinically appropriate (Anders and Trautmann, 2013). Management is to stop the suspected injectable trigger when a serious reaction is suspected, treat anaphylaxis immediately when present, avoid further heparin exposure in suspected heparin-induced thrombocytopenia while initiating alternative anticoagulation as indicated, and involve dermatology when diagnosis is uncertain or necrosis is present (Kim et al., 2023; Patel and Khan, 2017). A practical triage algorithm for drug-induced cutaneous reactions is shown in Figure 2. Table 1 provides a morphology-based framework linking cutaneous phenotypes to common culprit drugs, systemic toxicity signals, and recommended immediate actions.

**TABLE 1:** Morphology-based phenotype map for drug-induced cutaneous reactions with typical latency and evidence basis.

Phenotype	Common culprit drug	Systemic toxicity signal	Immediate action
Morbilliform eruption *Latency: 2–21 days*	β-lactams, sulfonamides, AEDs, allopurinol	Usually self-limited; may herald DRESS if fever, facial edema, or eosinophilia is present	Stop drug if red flags; monitor for progression *[Evidence: observational studies*, *case series*, *pharmacovigilance]*
Urticaria/angioedema *Latency: minutes to hours*	Antibiotics, NSAIDs, radiocontrast, ACEi	Anaphylaxis risk; airway compromise with laryngeal edema	IM epinephrine if anaphylaxis; stop trigger; avoid ACEi in bradykinin-mediated angioedema *[Evidence: RCTs*, *guidelines*, *pharmacovigilance]*
SJS/TEN *Latency: 4–28 days*	AEDs, allopurinol, sulfonamides, β-lactams	Fluid/electrolyte loss, infection, respiratory compromise, multiorgan failure; mortality 10%–30%	Immediate drug withdrawal; burn-unit-style supportive care; SCORTEN scoring *[Evidence: registries*, *cohort studies*, *validated scoring (SCORTEN)]*
DRESS *Latency: 2–8 weeks*	AEDs, allopurinol, sulfonamides, antibiotics	Hepatitis, nephritis, pneumonitis, myocarditis, cytopenias	Immediate drug withdrawal; systematic organ screening over weeks *[Evidence: prospective registry (RegiSCAR)*, *cohort studies]*
AGEP *Latency: ∼48 h*	Antibiotics (esp. β-lactams, macrolides)	Usually self-limited; systemic involvement in subset	Stop suspected trigger; supportive care; escalate if hemodynamic instability *[Evidence: case series*, *consensus criteria (EuroSCAR)]*
Palpable purpura/CSV *Latency: days to weeks*	β-lactams, NSAIDs, many others	Renal involvement, GI hemorrhage, arthralgias	Stop suspected drug; urinalysis and Cr; rheumatology/nephrology if systemic *[Evidence: case series*, *expert consensus]*
Retiform purpura/necrosis *Latency: 3–10 days (agent-dependent)*	Warfarin, heparin, vasopressors	Thrombotic syndromes (HIT and WISN), DIC, catastrophic vascular occlusion	Stop anticoagulant; urgent hematology; HIT workup; alternative anticoagulation *[Evidence: case series*, *pharmacovigilance*, *guidelines (HIT)]*
Drug-induced BP/pemphigus *Latency: weeks to months*	DPP-4i, ICIs	Infection risk, protein/fluid loss, immunosuppression sequelae	Stop drug if feasible; biopsy with DIF; dermatology-guided immunosuppression *[Evidence: case series*, *systematic reviews]*
Linear IgA BD *Latency: 1–14 days*	Vancomycin	Context dependent; forces antimicrobial reassessment	Stop vancomycin; switch antimicrobial; biopsy with DIF *[Evidence: case series*, *pharmacovigilance]*
Phototoxicity/photoallergy *Latency: hours to weeks (cumulative)*	Tetracyclines, thiazides, amiodarone, voriconazole	Chronic photodamage; elevated SCC risk (esp. voriconazole in transplant)	Strict photoprotection; reassess long-term drug necessity *[Evidence: cohort studies*, *systematic reviews]*
Acneiform eruption *Latency: 1–3 weeks*	EGFRi, corticosteroids, lithium	On-target marker (EGFRi); dose limiting if severe	Early supportive care; preserve anticancer dosing when possible *[Evidence: RCTs (oncology)*, *systematic reviews]*
HFS/HFSR *Latency: days to weeks (dose dependent)*	Fluoropyrimidines; MKIs (sorafenib and sunitinib)	Dose-limiting epithelial toxicity; ulceration and treatment interruption	Dose delay/reduction; emollients, keratolytics; prevent progression *[Evidence: RCTs*, *meta-analyses]*
Mucocutaneous erosions *Latency: within treatment cycles*	Chemotherapy, TKIs, ICIs	Neutropenic infection risk; SJS/TEN if skin pain and detachment	Assess for SCAR; supportive care; infection and neutropenia triage *[Evidence: RCTs (oncology), case series]*

Abbreviations: AED, antiepileptic drug; ACEi, angiotensin-converting enzyme inhibitor; AGEP, acute generalized exanthematous pustulosis; BP, bullous pemphigoid; BSA, body surface area; Cr, creatinine; CSV, cutaneous small vessel vasculitis; DIC, disseminated intravascular coagulation; DIF, direct immunofluorescence; DPP-4i, dipeptidyl peptidase-4, inhibitor; DRESS, drug reaction with eosinophilia and systemic symptoms; EGFRi, epidermal growth factor receptor inhibitor; GI, gastrointestinal; HFS, hand-foot syndrome; HFSR, hand-foot skin reaction; HIT, heparin-induced thrombocytopenia; ICI, immune checkpoint inhibitor; IM, intramuscular; LFT, liver function test; MKI, multikinase inhibitor; NSAID, nonsteroidal anti-inflammatory drug; SCC, squamous cell carcinoma; SCORTEN, Score of toxic epidermal necrosis; SJS, Stevens–Johnson syndrome; TEN, toxic epidermal necrolysis; TKI, tyrosine kinase inhibitor; WISN, warfarin-induced skin necrosis.

## 5 Drug-class-centered signature phenotypes

In oncology, immune checkpoint inhibitors commonly produce inflammatory eruptions that act as an early surface readout of systemic immune activation, including vitiligo-like depigmentation, lichenoid dermatitis, and immune checkpoint inhibitor-associated bullous pemphigoid (Asdourian et al., 2022; Thakker et al., 2025; Guida et al., 2021; Geisler et al., 2020). These phenotypes flag higher risk for concurrent immune toxicities and usually warrant symptom-graded management with a low threshold for coordinated screening when systemic symptoms develop, while aiming to preserve anticancer benefit through early dermatologic control (Geisler et al., 2020; Li et al., 2019). Epidermal growth factor receptor inhibitors produce a characteristic acneiform follicular eruption that often correlates with exposure and clinical outcomes, so the management implication is not simply to stop therapy but to treat early and prevent dose-limiting severity (Gorji et al., 2022; Wacker et al., 2007; Kiyohara et al., 2013). Hand-foot syndrome and hand-foot skin reaction from fluoropyrimidines and multikinase inhibitors are classic dose-limiting toxicities for which the actionable step is rapid supportive care plus timely dose delay or reduction to prevent ulceration and treatment discontinuation (Kwakman et al., 2020; Chu et al., 2008).

Among antimicrobials, beta-lactams and sulfonamides frequently trigger morbilliform eruptions and urticaria that are often benign, yet they also account for a large share of antibiotic-associated SCARs, so escalation hinges on timing, fever, mucosal disease, skin pain, and laboratory evidence of organ injury (Lee et al., 2023; Frey et al., 2018; Mockenhaupt et al., 2008). Vancomycin has a signature autoimmune blistering phenotype, drug-induced linear IgA bullous dermatosis, which can mimic epidermal necrolysis and therefore should prompt urgent drug withdrawal and antimicrobial substitution while confirming diagnosis by biopsy and direct immunofluorescence (Chu et al., 2008; Pereira et al., 2016). In antiretroviral therapy, abacavir hypersensitivity is a paradigm of actionable visual toxicity prevention because prospective HLA-B*57:01 screening reduces clinically suspected hypersensitivity, and rechallenge is contraindicated (Mounzer et al., 2019). Nevirapine remains a recognized driver of SCARs in pharmacovigilance series, making early rash with systemic symptoms a stop signal with escalation for mucosal or blistering disease (Oshikoya et al., 2020; du Toit et al., 2021).

For anticonvulsants and psychiatric drugs, aromatic anticonvulsants are signature culprits for SCARs, and the HLA-B*15:02 association with carbamazepine-related SJS–TEN underpins risk-targeted prescribing and avoidance in at-risk populations (Tangamornsuksan et al., 2013; Chen et al., 2011). Lithium has recognizable acneiform and psoriasiform phenotypes that primarily signal adherence and quality of life risk but can drive systemic treatment changes when severe, so management often balances dermatologic therapy with psychiatric necessity (El-Mallakh et al., 2025; Jafferany, 2008).

In cardio metabolic care, amiodarone phototoxicity and blue-gray hyperpigmentation serve as visible markers of cumulative exposure that should trigger sun-avoidance counseling and reconsideration of long-term therapy when cosmetically or functionally significant (Jaworski et al., 2014; Ammoury et al., 2008). Glucagon-like peptide 1 receptor agonists most often cause injection-site reactions and occasional generalized hypersensitivity eruptions, so the management implications are site rotation, evaluation for allergy when generalized rash occurs, and discontinuation when reactions recur or escalate (Samhani et al., 2024; Taj et al., 2025). In rheumatology and biologics, tumor necrosis factor alpha inhibitors have a well-described paradoxical psoriasis phenotype that may require topical therapy, systemic psoriasis treatment, or switching to a different biologic class, depending on severity and underlying disease control (Li et al., 2019; Maronese et al., 2024). Janus kinase inhibitors can cause acneiform lesions that are usually manageable without stopping therapy but require anticipatory counseling and infection surveillance when lesions are severe (Ballanger et al., 2023; Honap et al., 2026). Interleukin 17 inhibitors have a signature mucocutaneous candidiasis signal that should prompt early recognition and antifungal treatment and may require reassessment in patients with gastrointestinal symptoms suggestive of inflammatory bowel disease events (Truong et al., 2021; Davidson et al., 2022). Table 2 presents signature cutaneous phenotypes organized by drug class, highlighting systemic implications and key management considerations for oncology, antimicrobial, neuropsychiatric, and rheumatologic agents.

**TABLE 2:** Drug-class signature phenotypes.

Drug class	Signature phenotype(s)	Systemic implication	Key management point
ICIs	Vitiligo-like depigmentation, lichenoid dermatitis, ICI-associated BP	Early marker of immune activation; higher risk for concurrent irAEs	Symptom-graded care; low threshold for extracutaneous irAE screening
EGFRi	Acneiform papulopustular eruption (face, scalp, trunk)	Pharmacodynamic exposure marker; correlates with clinical response	Early prophylaxis and treatment to preserve dosing
Fluoropyrimidines/MKIs	HFS/HFSR (palmoplantar erythema, dysesthesia, desquamation)	Dose-limiting toxicity; may require interruption or reduction	Emollients, keratolytics; timely dose modification
β-lactams/sulfonamides	Morbilliform eruption, urticaria; SCARs in minorities	Usually benign; escalate if mucosal, skin pain, or systemic signs	Stop if red flags; document for future avoidance
Vancomycin	Linear IgA bullous dermatosis (tense bullae, annular pattern)	Mimics SJS/TEN; requires antimicrobial switch	Stop vancomycin; confirm with biopsy + DIF
Nevirapine	Early rash; SCAR (SJS/TEN, DRESS)	High SCAR incidence in pharmacovigilance data	Stop signal if mucosal or blistering; escalate urgently
Abacavir	Hypersensitivity syndrome (rash, fever, GI, respiratory)	Rechallenge can be fatal	HLA-B*57:01 screening before prescribing; contraindicate re-exposure
Aromatic AEDs (CBZ, PHT, and LTG)	SCAR (SJS/TEN, DRESS)	Strong HLA associations; cross reactivity among aromatics	HLA-B*15:02/HLA-A*31:01 screening; avoid cross-reactive agents after a SCAR
Lithium	Acneiform and psoriasiform eruptions	Primarily adherence/quality of life (QoL) impact; rarely life-threatening	Balance dermatologic therapy with psychiatric necessity
TNFi	Paradoxical psoriasis (new-onset psoriasiform dermatitis)	May require biologic class switch if severe	Topical therapy first; switch TNFi or class if refractory
JAKi	Acneiform eruptions	Usually manageable without stopping therapy	Anticipatory counseling; infection surveillance if severe
IL-17i	Mucocutaneous candidiasis	Watch for GI symptoms suggesting IBD exacerbation	Early antifungal treatment; IBD reassessment if indicated

Abbreviations: AED, antiepileptic drug; BP, bullous pemphigoid; CBZ, carbamazepine; DIF, direct immunofluorescence; EGFRi, epidermal growth factor receptor inhibitor; GI, gastrointestinal; HFS, hand-foot syndrome; HFSR, hand-foot skin reaction; HLA, human leukocyte antigen; IBD, inflammatory bowel disease; ICI, immune checkpoint inhibitor; IL-17i, interleukin-17, inhibitor; irAE, immune-related adverse event; JAKi, Janus kinase inhibitor; LTG, lamotrigine; MKI, multikinase inhibitor; PHT, phenytoin; QoL, quality of life; SCAR, severe cutaneous adverse reaction; SJS, Stevens-Johnson syndrome; TEN, toxic epidermal necrolysis; TNFi, tumor necrosis factor inhibitor.

## 6 Timing, dose, and patient susceptibility

Latency is one of the most informative features when the skin is used as an early warning sign. Immediate reactions, including urticaria, angioedema, and anaphylaxis, usually occur within minutes to several hours after drug exposure, and this short window supports rapid trigger identification and urgent risk mitigation when systemic features appear (Brockow et al., 2023). Delayed exanthematous reactions typically evolve over days, with morbilliform drug eruptions often appearing about 2 days to 21 days after starting a culprit medication, and recurrence after re-exposure can occur sooner (Calle et al., 2023; Yan et al., 2025). SCARs also follow characteristic windows that help distinguish them from low-risk rashes. AGEP often begins rapidly, frequently within about 48 h of the causative medication (Calle et al., 2023; Moore et al., 2025). SJS–TEN most often begins within the first month after drug initiation, with many drugs clustering in a roughly 4–28-day interval (Valeyrie-Allanore et al., 2013; Kim et al., 2014). DRESS has a longer latency, typically 2 weeks to 6 weeks and sometimes longer, which is diagnostically useful when systemic deterioration occurs weeks into therapy (Calle et al., 2023).

Some phenotypes are largely dose related, while others are idiosyncratic. Phototoxic drug reactions are dose dependent with respect to both drug and light exposure, making counseling and photoprotection immediately actionable when the pattern is recognized (Di Bartolomeo et al., 2022; Hofmann and Weber, 2021). Hand-foot syndrome from fluoropyrimidines and related agents behaves like an exposure-linked toxicity that can worsen with ongoing dosing and improve with dose interruption or reduction, so early identification supports prevention of ulceration and treatment disruption (de With et al., 2023; Abushullaih et al., 2002). In contrast, severe immune-mediated reactions such as SJS–TEN and DRESS are generally not predictable from dose alone and are shaped by immune recognition and host factors (Calle et al., 2023; Kim et al., 2014).

Susceptibility is modified by patient context. Older age and polypharmacy increase the baseline risk of adverse drug events and complicate culprit identification, which is particularly relevant for cutaneous reactions in older adults (Carneiro et al., 2011; Kim et al., 2024). Renal dysfunction and a high starting dose increase the risk for allopurinol hypersensitivity and severe outcomes, illustrating how impaired clearance can convert standard prescribing into higher effective exposure (Yang et al., 2015; Chung et al., 2015; Alotaibi et al., 2024). In DRESS, herpesvirus reactivation is repeatedly linked to more severe or prolonged courses. Early recognition can justify closer organ monitoring and follow-up (Chan et al., 2024; Mizukawa and Shiohara, 2025).

Pharmacogenomics is most clinically used for a small set of high-risk gene–drug pairs. HLA-B*57:01 screening before abacavir reduces immunologically confirmed hypersensitivity and is integrated into clinical practice guidance and CPIC recommendations (Mallal et al., 2008; Martin et al., 2014). HLA-B*15:02 testing before carbamazepine in higher-prevalence ancestries reduces SJS–TEN risk, and CPIC guidance also addresses HLA-A*31:01 in relation to carbamazepine and oxcarbazepine reactions (Chen et al., 2011; Phillips et al., 2018). HLA-B*58:01 is strongly associated with allopurinol-induced SCARs and is supported by CPIC guidance for genotype-informed prescribing (Saito et al., 2016). Many additional associations are under study, such as HLA-B*13:01 for dapsone hypersensitivity, but routine screening is not yet widespread outside specific indications and settings (Zhang et al., 2013). Table 3 summarizes typical latency windows and recommended laboratory evaluations for major drug-induced cutaneous syndromes to guide early recognition and workup.

**TABLE 3:** Latency windows and key laboratory workup by syndrome.

Syndrome	Typical latency	Key laboratory workup
Immediate urticaria/anaphylaxis	Minutes to hours	Tryptase (within 1–2 h of event)
Morbilliform drug eruption	2–21* *days	CBC, LFTs, Cr, urinalysis if systemic symptoms present
AGEP	∼48 h (range 1–11* *days)	CBC (neutrophilia), LFTs, Cr
SJS/TEN	4–28* *days	SCORTEN variables (age, malignancy, HR, BUN, glucose, bicarbonate, BSA); LFTs, Cr, electrolytes
DRESS	2–8* *weeks	CBC with differential (eosinophilia, atypical lymphocytes), LFTs, Cr, urinalysis; troponin/BNP if cardiac symptoms; consider HHV-6/CMV/EBV
Drug-induced vasculitis	Days to weeks	Urinalysis (hematuria, proteinuria), Cr, skin biopsy with DIF
HIT with skin necrosis	5–10* *days (or sooner if prior heparin)	Platelet count, 4Ts score, HIT antibody (PF4), coagulation panel
Warfarin-induced skin necrosis	3–6* *days after initiation	PT/INR, protein C/S levels (if feasible before warfarin started)

Abbreviations: AGEP, acute generalized exanthematous pustulosis; BNP, B-type natriuretic peptide; BSA, body surface area; BUN, blood urea nitrogen; CBC, complete blood count; CMV, cytomegalovirus; Cr, creatinine; DIF, direct immunofluorescence; DRESS, drug reaction with eosinophilia and systemic symptoms; EBV, Epstein-Barr virus; HHV-6, human herpesvirus 6; HIT, heparin-induced thrombocytopenia; HR, heart rate; INR, international normalized ratio; LFT, liver function test; PF4, platelet factor 4; PT, prothrombin time; SCORTEN, Score of toxic epidermal necrosis; SJS, Stevens-Johnson syndrome; TEN, toxic epidermal necrolysis.

## 7 From rash to risk: a practical diagnostic and triage framework

At the bedside, the priority is separating high-risk, time-critical phenotypes that require immediate drug withdrawal and urgent evaluation from lower-risk or self-limited eruptions that may be managed with symptomatic care and watchful monitoring. Triage works best when the first pass is visual pattern plus clock plus danger signs. The start should be anchoring morphology and then mapping latency to mechanism. Minutes to hours with wheals or angioedema points to an immediate mediator-driven risk, while days to weeks with a morbilliform eruption raises concern for delayed hypersensitivity concern, and a latency of 2 weeks to 8 weeks with facial edema or systemic symptoms should shift suspicion toward DRESS (Justiniano et al., 2008). When epidermal tenderness, dusky targetoid change, blistering, or any mucosal erosion is present, the presentation should be treated as potential epidermal necrolysis until proven otherwise with application of SCORTEN early to frame prognosis and level-of-care needs (Bastuji-Garin et al., 2000).

The essential screenings are fever, facial edema, mucosal disease, skin pain, necrosis, hypotension, tachycardia out of proportion, or rapidly expanding involvement. The minimum lab set for any eruption with systemic symptoms or high-risk morphology is complete blood count with the differential including eosinophils, liver enzymes with bilirubin, creatinine, urinalysis, and basic electrolytes to detect early organ involvement and fluid and electrolyte imbalance (Dagnon da Silva et al., 2023; Martinez-Cabriales et al., 2019; Schunkert and Divito, 2021). Creatine kinase can be added when pain is prominent or myositis is possible, and troponin with electrocardiography can be added when chest symptoms, marked tachycardia, syncope, or biomarker concern suggest myocarditis risk in DRESS (Bourgeois et al., 2012; Radovanovic et al., 2022). Using structured scoring reduces diagnostic drift; RegiSCAR can be used for DRESS severity attribution, and EuroSCAR criteria provide support for AGEP because both syndromes can look like common exanthems early yet diverge in monitoring and escalation needs (Sasidharanpillai et al., 2022; De et al., 2018).

Biopsy is most useful when the morphology is high risk, the diagnosis is uncertain, or management hinges on distinguishing between competing syndromes such as early SJS and TEN versus AGEP or autoimmune blistering disease (Mockenhaupt, 2009). In suspected cutaneous small vessel vasculitis, biopsy is a core diagnostic step because clinicopathologic discordance is common, and adding a second specimen for direct immunofluorescence increases yield and mechanistic classification (Alpsoy, 2022; Nandeesh and Tirumalae, 2013). Timing matters, with the highest direct immunofluorescence positivity when sampling is early in lesion evolution (Alpsoy, 2022).

Admission thresholds are guided by physiology and barrier failure. Any suspected SJS or TEN, extensive blistering, significant mucosal involvement, hemodynamic instability, inability to maintain oral intake, rapidly progressive purpura with pain or necrosis, or laboratory evidence of hepatitis, nephritis, cytopenias, or cardiac involvement warrants urgent inpatient care, with burn-unit-style management for epidermal necrolysis phenotypes (Bastuji-Garin et al., 2000).

## 8 Visual risk signals: measurement, documentation, and digital opportunities

Reliable visual risk signals start with standardized language for morphology, primary lesion type, secondary change, and surface anatomy terms so that different clinicians describe the same eruption in the same way across settings (Nast et al., 2016). Distribution should be recorded with precise anatomic labels, laterality, and regional qualifiers because location patterns often narrow differential diagnosis and improve follow-up comparability when images are not available (Navarrete-Dechent et al., 2020).

Extent should be quantified as body surface area using a consistent method, and for epidermal necrolysis phenotypes, this measurement is clinically decisive because consensus classification separates SJS–TEN by percent detachment thresholds (Bastuji-Garin et al., 1993). For toxic epidermal necrolysis, severity documentation should include SCORTEN variables alongside skin findings because this score was developed and validated to predict mortality and supports early escalation decisions tied to outcomes (Bastuji-Garin et al., 2000).

For DRESS, a structured case definition using the RegiSCAR scoring system improves consistency and helps distinguish true syndrome cases from common exanthems that do not carry the same organ monitoring burden (Sasidharanpillai et al., 2022).

Clinical photography adds value when it is reproducible, so acquire close-up views with stable lighting, fixed distance, and a scale marker, while considering cross-polarized and white light pairs, when feasible, to reduce glare and improve identification of erythema and texture (Oh et al., 2022). Because image sharing can expose identifiable information, consent should be explicit, purpose-specific, and paired with secure storage and restricted-access workflows that match modern secondary use risks in clinical care and education (Shinkai et al., 2023). Real-world audits of referral photos show that image quality and data handling are variable, supporting the need for minimum technical standards and governance before photographs are relied on for triage decisions (Castro Almeida et al., 2025). The clinical triage framework described above depends on accurate, timely pattern recognition, and digital tools can extend this capability beyond specialist settings by enabling remote assessment, standardized documentation, and, prospectively, automated phenotype detection.

Teledermatology can function as an early warning layer when bedside expertise is limited, and evidence syntheses in skin cancer pathways show it can support referral decisions, although performance varies by setting and lesion type (Chuchu et al., 2018). In inpatient care, store-and-forward teledermatology has been associated with improved management for high-risk bullous eruptions, illustrating how rapid image-based review can accelerate recognition of dangerous drug phenotypes (Shah and English, 2024). Computer vision systems trained on clinical images can reach specialist-level performance for selected classification tasks and may eventually support automated alerts when new drug-linked morphologies appear during therapy (Esteva et al., 2017; Choy et al., 2023). Limitations are material because dermatology models show performance drops and bias with underrepresented skin tones and dataset shifts, so any early warning deployment needs external validation, fairness evaluation, and human oversight to prevent systematic harm (Choy et al., 2023; Benčević et al., 2024).

## 9 Management principles and stop/continue decisions

In suspected SJS or TEN, immediate cessation of all temporally culprit drugs is an absolute rule because earlier withdrawal is associated with lower mortality (Garcia-Doval et al., 2000). When blistering or erosions develop during a drug eruption, prompt withdrawal remains the priority action even before confirmatory testing because delay can translate into preventable deaths (Miliszewski et al., 2016). In DRESS, the core management decision is immediate withdrawal of the offending drug followed by structured surveillance for evolving organ involvement over subsequent weeks (Wang et al., 2024). For low-risk morbilliform eruptions without fever, mucosal disease, skin pain, facial edema, or laboratory signals of organ injury, clinicians may continue essential therapy while treating symptoms and reassessing frequently for trajectory change (Broyles et al., 2020).

Symptom control for uncomplicated exanthems and urticaria relies on oral antihistamines and topical corticosteroids, with escalation only when systemic features or high-risk morphology appear (Khan et al., 2022). Systemic corticosteroids are commonly used in DRESS with significant organ involvement or clinical deterioration, with careful tapering to reduce relapse risk while monitoring hepatic, renal, hematologic, and cardiopulmonary parameters (Wang et al., 2024).

In SJS and TEN, supportive care remains the universally accepted foundation because comparative evidence for systemic immunomodulators is heterogeneous and inconsistent across meta-analyses (Chang et al., 2022). Cyclosporine has shown a mortality signal in pooled analyses and is used in select settings where contraindications are absent, and expert teams can monitor infection and renal risk (Ng et al., 2018). Intravenous immunoglobulin remains controversial due to variable outcome data and is generally considered only within protocolized care alongside meticulous supportive management (Chang et al., 2022).

After SJS, TEN, or DRESS, re-exposure through drug challenge or desensitization is contraindicated because recurrence can be catastrophic (Broyles et al., 2020; Khan et al., 2022). For aromatic antiepileptic drug rashes, cross reactivity is clinically meaningful, so alternative selection should avoid related aromatic agents when the initial reaction was severe or systemic (Alve et al., 2008). Future prevention depends on precise documentation of the culprit drug, timing, phenotype, severity, and organ involvement, plus formal reporting to pharmacovigilance systems because spontaneous reports are a major input for detecting rare SCAR signals and high-risk drug patterns (Mwakawanga et al., 2023). Hospital-based pharmacovigilance studies in cutaneous adverse drug reactions show that systematic reporting can identify preventable contributors and support safer prescribing through feedback loops (Sharma et al., 2019).

## 10 Special populations

Pregnancy adds dual patient stakes and different baseline differentials, so a suspected SCAR is managed with the same urgent stop rules and supportive priorities while incorporating obstetric assessment for fetal status and delivery planning (Sharma et al., 2020). Systematic reviews and population data suggest SJS and TEN during pregnancy are uncommon and often have favorable maternal outcomes, although higher preterm birth risk has been reported, so early recognition and multidisciplinary care remain essential (Sharma et al., 2020; Wasuwanich et al., 2024). HIV coinfection and antiretroviral exposure can shape pregnancy risk, with nevirapine repeatedly linked to SJS and TEN and higher baseline CD4 counts described as a risk context in several cohorts and reviews (Knight et al., 2015).

In pediatrics, delayed hypersensitivity syndromes can present with less classic prodrome and faster trajectory to systemic involvement, so clinicians often apply structured criteria and a low threshold laboratory screening when fever or facial edema accompanies a drug eruption (Kim et al., 2020; Chiriac et al., 2024). A systematic review of pediatric DRESS found liver involvement to be common and overall prognosis frequently good with treatment, but it also documented serious complications that justify standardized monitoring after culprit withdrawal (Kim et al., 2020).

In older and frail adults, polypharmacy, multimorbidity, and age-related pharmacokinetic changes increase the probability of drug eruptions and complicate culprit attribution, so deprescribing-focused reconciliation is often part of rash management (Carneiro et al., 2011; Woo et al., 2020). Frailty is independently associated with higher vulnerability to adverse drug events, which supports more conservative stop or continue decisions and earlier escalation when systemic symptoms accompany a rash (Dorj et al., 2023).

In immunocompromised and transplant populations, cutaneous toxicity may signal cumulative exposure and increased malignancy risk rather than classic immune hypersensitivity, as illustrated by cohort evidence linking voriconazole exposure to higher keratinocyte carcinoma risk in lung transplant recipients (D’Arcy et al., 2020). In people living with HIV, severe cutaneous reactions remain clinically relevant, with pediatric series documenting nevirapine-associated SJS and reinforcing the need for rapid drug substitution when mucosal or painful dusky lesions appear (du Toit et al., 2021).

In darker skin tones, erythema can be less visible, which can delay recognition of inflammatory severity and contribute to diagnostic disparities, so clinicians should deliberately assess warmth, edema, dyschromia, and textural change rather than relying on redness alone (Forsyth et al., 2025; Taylor, 2023). Adjunct tools such as dermoscopy can help as pigmentary surrogates of inflammation may be more apparent than vascular color change in richly pigmented skin, improving detection when erythema is muted (Bhat and Jha, 2021).

## 11 Knowledge gaps and research agenda

A central gap is that most drug eruption phenotypes are treated as categorical syndromes rather than validated predictive risk signals with quantified sensitivity, specificity, and positive predictive value for organ toxicity, which limits how confidently clinicians can act on early morphology alone (Blumenthal et al., 2024). Bridging that gap requires prospective, deeply phenotyped cohorts that capture standardized morphology, timing, drug exposure windows, and sequential laboratory and imaging endpoints so that phenotype can be linked to incident hepatic, renal, pulmonary, hematologic, and cardiac outcomes with time-to-event analyses (King et al., 2024).

For DRESS, current cohorts show substantial heterogeneity by culprit drug and organ patterning, but much of the literature remains retrospective and vulnerable to misclassification, underscoring the need for prospective designs that anchor adjudication to RegiSCAR definitions while collecting objective outcome data (Blumenthal et al., 2024; King et al., 2024). For epidermal necrolysis, systematic reviews document marked heterogeneity in reported outcomes, definitions, and timing, which impairs comparison of immunomodulatory strategies and supportive care models across studies (Libson et al., 2024; Dobry et al., 2022). A practical research agenda, therefore, includes harmonized outcome sets that go beyond mortality to capture time to re-epithelialization, infection, ocular sequelae, patient-reported symptoms, and long-term function, aligned with international consensus efforts (Ingen-Housz-Oro et al., 2025).

Imaging standards are a second bottleneck because prediction models and multicenter cohorts cannot generalize when clinical photographs vary in lighting, framing, scale, and metadata, so protocolized acquisition and training standards from teledermatology guidance should be adapted for drug toxicity research (Quigley et al., 2015; Ashique et al., 2015). Digital triage and computer vision offer a route to earlier detection and wider access, but safe implementation depends on datasets that include diverse skin tones, real-world inpatient images, and labels that reflect clinician consensus and biopsy where appropriate (Tjiu and Lu, 2025; Weir et al., 2025). Multiple evaluations show performance drops and fairness gaps for darker skin tones and non-specialist settings, making external validation, subgroup reporting, and deployment monitoring mandatory rather than optional (Benčević et al., 2024). Near-term priorities include standardized skin tone labeling methods, transparent reporting of dataset composition, and calibration strategies that minimize false reassurance in underrepresented groups while maintaining acceptable false alert rates in routine care (Tjiu and Lu, 2025; Weir et al., 2025).

## 12 Conclusion

The most actionable drug-related skin signs for systemic toxicity are those that predict rapid deterioration or occult organ injury. Skin pain, dusky or targetoid change, blistering, and any mucosal erosion are stop signals for possible epidermal necrolysis and should trigger immediate culprit withdrawal, urgent admission-level supportive care, and early specialty coordination. Facial edema with a widespread eruption plus fever, lymphadenopathy, eosinophilia, or atypical lymphocytosis should trigger a DRESS pathway with prompt drug cessation and systematic screening for hepatic, renal, pulmonary, hematologic, and cardiac involvement. Palpable purpura, retiform purpura, livedo racemosa, and evolving necrosis should trigger urgent evaluation for vasculitis, thrombosis, and coagulopathy with rapid escalation when renal, gastrointestinal, or hemodynamic features are present.

A practical framework is phenotype to mechanism to systemic risk to action, using morphology, timing, and red flags to decide whether to stop or continue and to select a minimal but targeted evaluation set. Progress depends on standardized documentation, reproducible imaging, and multidisciplinary pathways linking dermatology, pharmacy, allergy, critical care, and the primary treating service to reduce delays and prevent recurrence.
